# Supplementary material for: Odd-paired is a pioneer-like factor that coordinates with Zelda to control gene expression in embryos
Source: eLife. 2020 Jul 23;9:e59610. doi: 10.7554/eLife.59610 (PMC7417190; doi:10.7554/eLife.59610)
Supplement: Figure 2—source data 1. [file elife-59610-fig2-data1.docx]

**Figure 2 – source data 1**

**I. Opa-only II. Zld-only III. Opa-Zld overlap**

**Opa-like 1e-522 (15.97%)**  n.f. **1e-43 (4.54%)**

**Zelda-like** n.f.  **1e-2833 (55.03%) 1e-240 (15.05%)**

**Trl-like** 1e-12 (6.78%) 1e-23 (4.45%) **1e-76 (10.56%)**

**Dref-like 1e-119 (6.08%)** n.f 1e-19 (4.13%)

**Cad-like** n.f. **1e-63 (14.18%)**  n.f.

**Unknown 1 1e-117 (5.93%)**  n.f.  **1e-33 (2.99%)**

**Run-like** 1e-22 (2.51%) n.f. n.f.

**Figure 2 – source data 1. Significance and abundance of motifs for known transcription factors found by HOMER within the three corresponding sets of peaks.** Each set of peaks (i.e. Opa-only, Zld-only, and Opa-Zld overlap called by comparison of Opa (3h) and Zld nc13-nc14 ChIP-seq data) was independently analyzed by HOMER, and binding site consensus sequences defined independently for the listed transcription factors. If no motif for a certain transcription was designated significant by HOMER within a particular set of peaks, designated not found (n.f.). P-values are shown for enrichment of consensus binding sites within each class; abundance shown in parentheses. Data for a subset of sites are shown in Figure 2.
