# Supplementary material for: Odd-paired is a pioneer-like factor that coordinates with Zelda to control gene expression in embryos
Source: eLife. 2020 Jul 23;9:e59610. doi: 10.7554/eLife.59610 (PMC7417190; doi:10.7554/eLife.59610)
Supplement: Figure 3—source data 2. [file elife-59610-fig3-data2.docx]

**Figure 3 – source data 2**

| Gene category | Genes up-regulated in *sh_Opa* (red; Fig. 3D) | Genes down-regulated in *sh_Opa*  (blue; Fig. 3D) |
| --- | --- | --- |
| All | **350** | **667** |
| ChIP Opa only | **128 (36.6%)**  Ex. *rca1, smc2, chd3, DNaseII* | **246 (36.8%)**  Ex. *CG3176,atf6* |
| ChIP Opa & Zld | **147 (42.2%)**  Ex. *ubi-p5E, eve* | **199 (29.8%)**  Ex. *Z600, bro* |
| Both ChIP Opa only and ChIP Opa & Zld | **224 (64.0%)**  Ex. *doc3, doc2* | **366 (54.9%)**  Ex. *Ih, sog* |

**Figure 3 – source data 2.** Differentially expressed genes identified by RNA-seq that are associated with Zld and/or Opa ChIP-seq defined occupancy with representative examples (Ex.) for each class (also noted in Fig. 3D). Individual genes can be associated with multiple peaks and thus can contain both Opa_only and Opa&Zld peak designations.
