# Supplementary material for: Odd-paired is a pioneer-like factor that coordinates with Zelda to control gene expression in embryos
Source: eLife. 2020 Jul 23;9:e59610. doi: 10.7554/eLife.59610 (PMC7417190; doi:10.7554/eLife.59610)
Supplement: Transparent reporting form [file elife-59610-transrepform.docx]

***eLife’s* transparent reporting form**

We encourage authors to provide detailed information *within their submission* to facilitate the interpretation and replication of experiments. Authors can upload supporting documentation to indicate the use of appropriate reporting guidelines for health-related research (see [EQUATOR Network](about:blank)), life science research (see the BioSharing Information Resource), or the [ARRIVE guidelines](about:blank) for reporting work involving animal research. Where applicable, authors should refer to any relevant reporting standards documents in this form.

If you have any questions, please consult our Journal Policies and/or contact us: [editorial@elifesciences.org](about:blank).

**Sample-size estimation**

- You should state whether an appropriate sample size was computed when the study was being designed
- You should state the statistical method of sample size computation and any required assumptions
- If no explicit power analysis was used, you should describe how you decided what sample (replicate) size (number) to use

Please outline where this information can be found within the submission (e.g., sections or figure legends), or explain why this information doesn’t apply to your submission:

Sample sizes were determined by experience. Specifically, for in situ hybridization n=5 per genotype, for live imaging experiments n=3, for ChIP-seq n=3 and for ATAC-seq n=3. Sample size is indicated in figure legends or directly in the figure.

**Replicates**

- You should report how often each experiment was performed
- You should include a definition of biological versus technical replication
- The data obtained should be provided and sufficient information should be provided to indicate the number of independent biological and/or technical replicates
- If you encountered any outliers, you should describe how these were handled
- Criteria for exclusion/inclusion of data should be clearly stated
- High-throughput sequence data should be uploaded before submission, with a private link for reviewers provided (these are available from both GEO and ArrayExpress)

Please outline where this information can be found within the submission (e.g., sections or figure legends), or explain why this information doesn’t apply to your submission:

Replicate information is included in the materials and methods as well as in the figure legends. No outliers were encountered. GEO accession number can be found within the manuscript [GEO accession number SuperSeries GSE153329; ChIP-seq and singled-end ATAC-seq (GSE140722), and RNA-seq and paired-end ATAC-seq data access (GSE153328)]. The codes for RNA-seq, Opa ChIP-seq and ATAC-seq processing (alignment and peak calling) were uploaded to github: <https://github.com/caltech-bioinformatics-resource-center/Stathopoulos_Lab>)

**Statistical reporting**

- Statistical analysis methods should be described and justified
- Raw data should be presented in figures whenever informative to do so (typically when N per group is less than 10)
- For each experiment, you should identify the statistical tests used, exact values of N, definitions of center, methods of multiple test correction, and dispersion and precision measures (e.g., mean, median, SD, SEM, confidence intervals; and, for the major substantive results, a measure of effect size (e.g., Pearson's r, Cohen's d)
- Report exact p-values wherever possible alongside the summary statistics and 95% confidence intervals. These should be reported for all key questions and not only when the p-value is less than 0.05.

Please outline where this information can be found within the submission (e.g., sections or figure legends), or explain why this information doesn’t apply to your submission:

The RNA-seq, ChIP-seq and ATAC-seq background modeling can be found in the Methods section. Plots and heatmaps, including statistical tests that were used for the analysis clearly indicate the sample sizes and either are described in the Methods and/or occasionally in the Figure legends. Exact p-values are provided for RNA-seq and ChIP-seq analyses. Additional description of the analysis for each method is provided at the GitHub page provided above.

(For large datasets, or papers with a very large number of statistical tests, you may upload a single table file with tests, Ns, etc., with reference to sections in the manuscript.)

**Group allocation**

- Indicate how samples were allocated into experimental groups (in the case of clinical studies, please specify allocation to treatment method); if randomization was used, please also state if restricted randomization was applied
- Indicate if masking was used during group allocation, data collection and/or data analysis

Please outline where this information can be found within the submission (e.g., sections or figure legends), or explain why this information doesn’t apply to your submission:

Experimental groups were defined by genotype with wildtype being represented by wt. Randomization was not performed.

**Additional data files (“source data”)**

- We encourage you to upload relevant additional data files, such as numerical data that are represented as a graph in a figure, or as a summary table
- Where provided, these should be in the most useful format, and they can be uploaded as “Source data” files linked to a main figure or table
- Include model definition files including the full list of parameters used
- Include code used for data analysis (e.g., R, MatLab)
- Avoid stating that data files are “available upon request”

Please indicate the figures or tables for which source data files have been provided:

We’ve put codes online here:

[https://github.com/brianpenghe/Stathopoulos_Lab](about:blank)

Annotated peaks lists are provided as additional data source files.
